# Analysis of Cell Dielectrophoretic Properties Using Isomotive Creek-Gap Electrode Device

**DOI:** 10.3390/s24237681

**Published:** 2024-11-30

**Authors:** Shigeru Tada, Noriko Sato

**Affiliations:** 1 National Defense Academy, Yokosuka 239-8686, Japan; 2 Graduate School of Applied Science and Engineering, National Defense Academy, Yokosuka 239-8686, Japan; pikapon927@gmail.com

**Keywords:** dielectrophoresis, cell manipulation, isomotive movement of cell, Clausius–Mossotti factor, AC nonuniform electric field

## Abstract

Various types of dielectrophoresis (DEP) cell separation devices using AC electric fields have been proposed and developed. However, its capability is still limited by a lack of quantitative characterization of the relationship between frequency and force. In the present study, this limitation was addressed by developing a method capable of fast and accurate quantification of the dielectric properties of biological cells. A newly designed Creek-gap electrode device can induce constant DEP forces on cells, realizing the isomotive movement of cells suitable for DEP analysis. The real number part of the Clausius–Mossotti (CM) factor of cells, Re(β), was obtained by simple cell velocimetry together with the numerical three-dimensional (3D) electric field analysis. Human mammary cells, MCF10A, and its cancer cells, MCF7 and MDAMB231, were used as model cells to evaluate the capability of the proposed device. The estimation of Re(β) using the Creek-gap electrode device showed good agreement with previously reported values. Furthermore, the thermal behavior of the Creek-gap electrode device, which is crucial to cell viability, was investigated by adopting micro laser-induced fluorescence (LIF) thermometry using Rhodamine B. The temperature rise in the device was found to be approximately several degrees Celsius at most. The results demonstrate that the proposed method could be a powerful tool for fast and accurate noninvasive measurement of the DEP spectrum and the determination of the dielectric properties of biological cells.

## 1. Introduction

In the field of biomedical engineering, the principle of dielectrophoresis (DEP) in A.C. electric fields has been attracting attention as a promising means of high-throughput cell separation and has been widely used to manipulate [1,2], separate [3,4], and characterize [5,6] biological cells. DEP is an electromechanical phenomenon in which polarizable but electrically uncharged particles subjected to a nonuniform electric field move in the direction of the gradient of the electric field. The driving force of cell movement is called the DEP force. DEP relies on the polarization effect exerted on cells in the electric field. Polarization effects are caused by differences in dielectric properties between cells and the suspension medium, and the resulting movement of cells in response to the electric field varies widely depending on the field frequency. Because the strength of the DEP force is proportional to the gradient of the square of the electric field, $\nabla E^{2}$, a reduction in the length scale of the device will significantly increase the DEP force. This ability to favorably scale the DEP force according to the size of the device makes DEP very suitable for manipulating and separating cells in microfluidic systems. Therefore, the most common and prevalent DEP cell separation devices have micro parallel-plate channel structures in which a pair of counter-interdigitated electrodes are installed on the bottom face [7,8,9]. In this type of cell separation device, cells are captured at the edges of the electrode, where $\nabla E^{2}$ is maximal. This unique feature of DEP has been widely utilized for the separation of subpopulations of cells [10,11,12,13,14], including live and dead cells [15], cancer sublines [16,17], and circulating tumor cells [18,19].

Although most DEP applications have focused primarily on cell separation and concentration, there is still unexplored potential for the application of high-throughput DEP to single-cell analysis. DEP can accurately measure the response of a single cell to an electric field. This allows individual cells to be measured without interference from other surrounding cells. Certainly, the nonuniform nature of DEP force is suitable for separation applications; however, conversely, this becomes the disadvantage of previously proposed DEP devices from the perspective of quantified cell analysis. A uniform DEP force field would allow accurate quantification of cell movement by determining the dielectric properties of the cell. For analytical purposes, $\nabla E^{2}$ should be constant throughout the measurement site. A DEP system in which $\nabla E^{2}$ is constant is termed isomotive DEP (isoDEP) and was first demonstrated by Pohl [20].

In previous studies on isoDEP, several device designs have been proposed to distribute DEP forces more effectively so that the cells undergo constant movement [21,22,23,24]. However, other than the device by Allen et al. [22], analysis was possible only in restricted regions of devices, and in many cases, generation of extremely strong electric fields or high-voltage applications that could affect cell viability and device function were required. The upper limit of the electric field strength used for cell separation is considered to be ~2 kV/cm, which is the threshold value at which cells are destroyed, as reported by Yuhao et al. [25]. Since the electrode spacing in cell separation experiments is generally ~100 μm in most cases, using the above threshold value, the upper limit of voltage would be ~20 V. Therefore, this value is assumed to be the high voltage in this study. The minimum electrode spacing of the device in this study is 100 μm, and the applied voltage is 7.1 V ($=10/\sqrt{2}$) in RMS, which does not correspond to a high voltage. To solve this problem, we developed an analytical DEP device capable of measuring negative DEP (n–DEP) forces induced on individual cells noninvasively, simply, and with low voltage application [26]. The real number part of the Clausius–Mossotti (CM) factor of cells, Re(β), is deduced from the velocimetry of cells moving through the channel of a Creek-gap electrode device, in which an electric field with constant $\nabla E^{2}$ is achieved. In a previous report [26], the magnitude of the DEP force acting on human living cells (MCF10A, HeLa) was evaluated using the proposed device, and the relationship between frequency and Re(β) was obtained. However, the DEP spectrum (Re(β) vs. frequency) did not agree very well with the theoretical values for a wide range of frequency values. Sensitivity analysis of Re(β) was also performed, and mass density of the cell, friction force between the cell and surface of the electrode substrate, and cell diameter were found to be the dominant parameters of the proposed method. In this study, to resolve the issues with the proposed device, the main focus was on the accurate evaluation of the values of the dominant parameters, and significant improvements were made to the analytical method, including more accurate estimation of mass density and development of a device capable of accurately measuring the coefficient of the kinetic friction. Simultaneously, the temperature rise in the device due to internal heat generation was investigated to assess the device reliability and effects on cells using microscale laser-induced fluorescence (micro LIF) thermometry.

In this paper, first, the basic principle of measurement, including the design rules of the Creek-gap electrode device, is briefly introduced. The DEP spectrum of model cells (human mammary cells) is then reported from the measured velocities of moving cells over a range of field frequencies, and the results are compared with those of conventional electrorotation. The dielectric properties of individual cells are then extracted using the best fit of the experiment to the theory. Finally, the reliability and accuracy of the measurement and operation performance of the proposed device, including its thermal behavior, are examined and discussed.

## 2. Creek-Gap Electrode Device

### 2.1. Principle of Measurement

**Figure 1:**
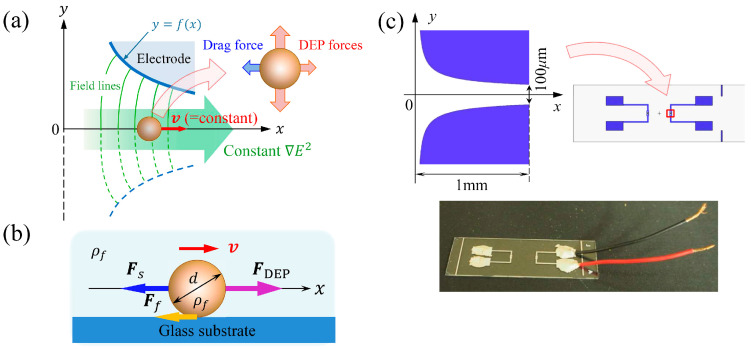
(**a**) Concept of the Creek-gap electrode device. The electric field generated by a pair of fan-shaped electrodes enables the isomotive motion of a single cell along the channel of the device. (**b**) When a single cell travels with a constant velocity, the negative dielectrophoretic force $F_{DEP}$ and the sum of the two resistant forces, Stokes drag force $F_{s}$ and friction force $F_{f}$, are balanced with each other. (**c**) (Top) Design of the Creek-gap electrode device, configured with a pair of planar thin metal electrodes with a creek (river mouth)-shaped channel between them. Electrodes were fabricated by standard photolithography. (Bottom) Photograph of the fabricated Creek-gap electrode device. Lead wires were bonded to the electrodes using electrically conductive 2-part resins.

As shown in Figure 1a, a single spherical cell of mass density and diameter, $\rho_{c}$ and *d*, respectively, travels along the x-axis with a constant velocity v in a suspension medium of mass density $\rho_{f}$. The movement of the cell is generated by the n–DEP force $F_{DEP}$ induced on the cell under the exposure of a nonuniform AC electric field E. The components of $F_{DEP}$ perpendicular to the direction of cell movement cancel each other, and only the component of $F_{DEP}$ parallel to the centerline of the channel contributes to the induction of cell movement. In addition to $F_{DEP}$, the Stokes drag force $F_{s}$ due to hydraulic resistance and friction force $F_{f}$ between the cell and bottom face of the electrode substrate also act on the cell in the opposite direction (−x) of the cell movement (Figure 1b). Although the frictional force is thought to be caused by the microscopic structure of the cell membrane surface and the glass surface, such as adhesive forces, electrostatic forces exist between them [27], but the details are not known.

The DEP force induced on the cell is defined as follows:

(1) $$F_{DEP}=2\pi \epsilon_{0}\epsilon_{f}{\left(\frac{\;d\;}{2}\right)}^{3}Re\left(\beta \right)\nabla {E_{rms}}^{2},$$

where $\epsilon_{0}$ is the vacuum electric permittivity and $\epsilon_{f}$ is the relative electric permittivity of the suspension medium. ∇ is the Nabla operator. $E_{rms}$ is the root mean square of the electric field E. The value Re(β) is the real number part of the CM factor β, defined as follows [28]:

(2) $$\beta =\frac{{\epsilon_{c}}^{*}-{\epsilon_{f}}^{*}}{\;{\epsilon_{c}}^{*}+{2\epsilon_{f}}^{*}\;},$$

where $\epsilon_{c}$ is the relative electric permittivity of the cell. The symbol * indicates that the value is complex, and the complex electric permittivity is written as follows:

(3) $$\epsilon^{*}=\epsilon +\frac{\sigma }{\;j\epsilon_{0}\omega \;},$$

where σ is the electric conductivity of materials, ω=2πf, f is the electric field frequency, and $j=\sqrt{-i\;}$. When a single cell travels with a constant velocity, $F_{DEP}$ and the sum of $F_{s}$ and $F_{f}$ are balanced with each other. Thus, the equation of force balance is denoted as follows:

(4) $$F_{DEP}-F_{s}-F_{f}=0$$

where $F_{DEP}=\left|F_{DEP}\right|$, $F_{s}=\left|F_{s}\right|$, $F_{f}=\left|F_{f}\right|$. Equation (4) implies that the electric field E has a distribution such that $\nabla {E_{rms}}^{2}$ is kept constant along the x axis; thus, Re(β) is written using Equations (1) and (4) as follows:

(5) $$Re\left(\beta \right)=\frac{F_{s}+F_{f}}{\;2\pi \epsilon_{0}\epsilon_{f}{\left(\frac{\;d\;}{2}\right)}^{3}\left|\nabla {E_{rms}}^{2}\right|\;}.$$

In Equation (5), the Stokes drag force $F_{s}$ acting on a spherical particle of diameter d moving over a plate wall with velocity v can be obtained as [29]:

(6) $$F_{d}=\kappa v\;\;\;\;\left(\kappa =1.7\times 6\pi \frac{\;d\;}{2}\eta \right),$$

where $v=\left|v\right|$, η is the medium viscosity. The friction force $F_{f}$ acts between the cell and bottom face of the electrode substrate as follows:

(7) $$F_{f}={\frac{\;4\;}{3}\pi \left(\rho_{c}-\rho_{f}\right)\left(\frac{\;d\;}{2}\right)}^{3}\mu g,$$

where μ is the coefficient of the kinetic friction and g is the coefficient of acceleration of gravity. When $\rho_{c}$, $\rho_{f}$, and μ are known and the value of $\left|\nabla {E_{rms}}^{2}\right|$ along the x axis is kept constant, the Re(β) of the cell can be immediately obtained by measuring d and v.

### 2.2. Creek-Gap Electrode Design and Fabrication

The schematics of the Creek-gap electrode device are shown in Figure 1c. To generate the electric field ***E*** of constant value $\left|\nabla {E_{rms}}^{2}\right|$ along the x-axis direction, the device consisted of a pair of planar electrodes with a fan-shaped flow channel between them. The channel geometry was determined as follows. Assume that the value of $\left|\nabla {E_{rms}}^{2}\right|$ along the centerline of the channel equals a constant value a:

(8) $${\left|\nabla {E_{rms}}^{2}\right|}_{y=0}=a.$$

Because the shapes of the paired electrodes are symmetric about the x-axis, Equation (8) is rewritten as follows:

(9) $${\left|\frac{\partial }{\;\partial x\;}{E_{rms}}^{2}\right|}_{y=0}=a.$$

The integration of both sides of Equation (9) yields:

(10) $$\left|E_{rms}\right|=\sqrt{ax+b\;}.$$

where b is a constant and is determined by the boundary conditions specified later. Meanwhile, the magnitude of the electric field across the channel width can be approximated as follows:

(11) $$\left|E_{rms}\right|≅\frac{{\;V}_{rms}}{\;2y\;},$$

where 2y is the channel width across electrodes at an arbitrary position x. Therefore, the outline of the Creek-gap electrode y is denoted as a function of x:

(12) $$y=\frac{V_{rms}}{\;\sqrt{ax+b\;}\;}.$$

The Creek-gap electrode used in the experiment was an aluminum film with a thickness of 300 nm fabricated by standard photolithography at the Semiconductor Center of Kitakyushu Foundation for the Advancement of Industry, Science, and Technology. The shape of the Creek-gap electrode was determined using Equation (12) with the boundary conditions of y=1.0 mm at x=0 mm and y=50 μm at x=1.0 mm. The constants a and b obtained are as follows:

(13) $$\left\{\begin{array}{c} \;a=1.1\times 10^{11}\;{V_{rms}}^{2}\;\;\left(V^{2}/m^{3}\right)\\ \;\;b=2.5\times 10^{5}\;{V_{rms}}^{2}\;\;\;\;\left(V^{2}/m^{2}\right)\; \end{array}\right..$$

### 2.3. Electric Field Computation

**Figure 2:**
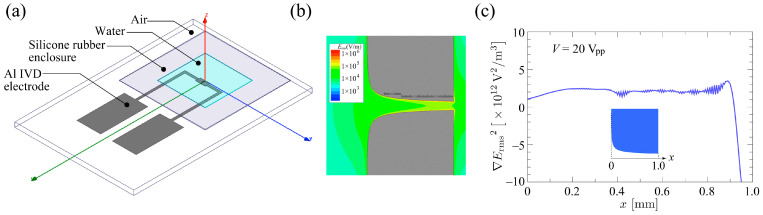
(**a**) Numerical simulation model of the Creek-gap electrode device. The geometry and dimensions of the model were exactly the same as those of the fabricated device. (**b**) Contours of the electric field distribution over the surface of the electrode substrate. (**c**) Profiles of $\nabla {E_{rms}}^{2}$ along the centerline of the channel. The prediction had a uniform distribution along the centerline of the channel except in regions on both ends of the channel.

In order to obtain the distribution of $\nabla {E_{rms}}^{2}$, a 3D numerical simulation of the electric field $E_{rms}$ in the Creek-gap electrode was performed. The commercial FEM software, ANSYS Maxwell v18.1, was used for the simulation. Figure 2a shows the numerical simulation model. The geometry and dimensions of the model comprising a glass substrate, metal electrodes, and silicone rubber spacer were exactly the same as those of the fabricated device. The number of FEM elements used was approximately 6,000,000. An applied voltage of $V_{rms}=10/\sqrt{2}$ V (V=20 V_pp_) was assumed. The relative permittivity of the suspension medium and the glass substrate used were $\epsilon_{f}=78.0$ and $\epsilon_{g}=6.0$, respectively. Figure 2b shows contours of the electric field distribution over the surface of the electrode substrate. The magnitude of the electric field reached its maximum at the position where the width of the channel was narrowest and reduced almost exponentially along the channel direction. Figure 2c shows the profile of the intensity of $\nabla {E_{rms}}^{2}$ along the centerline of the channel. The distribution of the $\nabla {E_{rms}}^{2}$ was almost uniform, except at both ends of the channel. Furthermore, the profile of $\nabla {E_{rms}}^{2}$ along the centerline has almost the same distribution as the profile in Figure 2c up to a height of about z~16 μm from the device surface, i.e., a height corresponding to the diameter of the cell. The value of the intensity of $\nabla {E_{rms}}^{2}$ on the plateau was $2.20\;\times \;10^{12}$ V^2^/m^3^, and this value was used for Re(β) estimation.

## 3. Experimental Setup and Method

### 3.1. Cell Preparation

**Figure 3:**
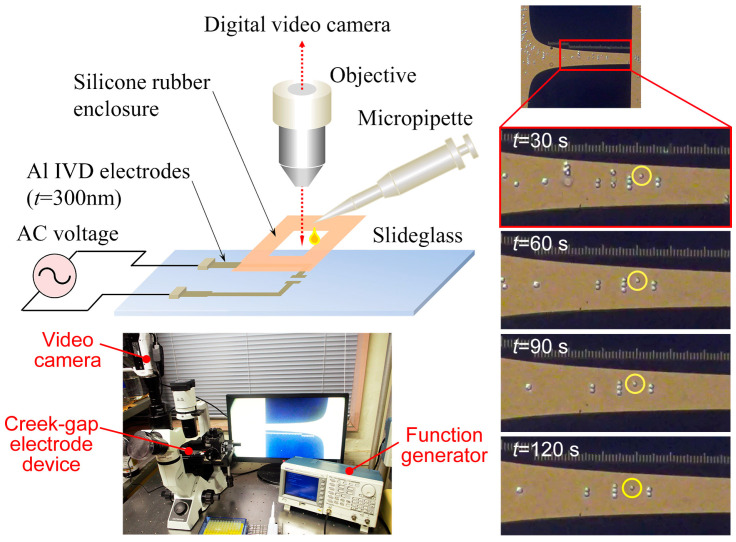
(**Left**) Schematic illustration and photograph of the experimental apparatus. The test section consisted of the Creek-gap electrode device and a silicone rubber enclosure. AC power was supplied by a function generator. (**Right**) Snapshots of the transient of MCF10A cell behavior in the channel of the Creek-gap electrode device at times t=30, 60, 90, and 120 s after the onset of the electric field application. All of the cells moved in the direction of the channel expansion with constant velocity.

Human mammary cells, MCF10A, and corresponding cancer cells, MCF7 and MDA–MB231, were used in the experiment. MCF10A cells were cultured in DMEM/F12 Ham’s Mixture (Thermo Fisher 1330032, Waltham, MA, USA) supplemented with 100 ng/mL cholera toxin (Sigma C8052, Saint Louis, MO, USA), 0.5 mg/mL hydrocortisone (Sigma H0135), 10 μg/mL human insulin (Sigma I9278), 20 ng/mL human epidermal growth factor (Thermo Fisher PHG0311L), and 5% horse serum (Thermo Fisher 16050122). MCF7 cells were cultured in MEM media supplemented with 10% fetal bovine serum (Gibco, Waltham, MA, USA), 0.1 mM MEM non-essential amino acid (Sigma M7145), 1 mM sodium pyruvate (Sigma S8636), and 0.584 g/L L–glutamine (Sigma G7513). MDA–MB231 cells were cultured in DMEM high glucose media (Sigma D5671) supplemented with 10% fetal bovine serum (Gibco) and 0.584 g/L L-glutamine (Sigma G7513). Cells were fed every 3 days and grown to confluence using ϕ60 culture dishes before subculturing. Cells of 8–10 passages were used for the experiments. For the experiment, cells were collected and suspended in the isotonic, non-electrolyte 300 mM mannitol solution (Sigma M4125). The electric conductivity of the solution was set to $\sigma_{f}=40$ mS/m by adding culture media. The appropriate amount was then pipetted into the square hole of the test section (Figure 3).

### 3.2. Setup and Procedure of Experiment Using Creek-Gap Electrode Device

Figure 3 shows the experimental setup. The test section of the setup consisted of the Creek-gap electrode device and a silicone rubber enclosure with a thickness of 0.1 mm on top. A 5 mm square hole was provided in the center of the silicone rubber enclosure for the cell suspension. A function generator (Tektronix, AFG3101, Beaverton, OR, USA) was used to generate an electric field in the device. The test section was mounted on the stage of a microscope (Olympus, CKX41, Tokyo, Japan) and the cell suspension was micropipetted into the square holes of the silicone rubber enclosure and then connected to the function generator. A sinusoidal AC voltage of V=20.0 V_pp_ and frequencies of f=10–50 kHz were applied to the device. This is because the DEP force induced in the cells became a repulsive force against the electrodes in the frequency range of 10–50 kHz of the AC electric field. The behaviors of cells were monitored using a digital video camera (Handycam, SONY, HANDYCAM HDR-CX680, Tokyo, Japan) installed on the video port of the microscope, and the recorded movies were used for analysis. The right-hand side of Figure 3 shows snapshots of the transient of MCF10A cell behavior in the channel of the Creek-gap electrode device at times t=30, 60, 90, and 120 s after the onset of the applied voltage with f=25 kHz. As shown in the figure, all cells moved in the direction of the channel expansion. Because cells exhibited n-DEP properties at this frequency, cells were repelled from the electrode and began to gather near the centerline of the channel immediately after the onset of the voltage application. After a short time, due to the action of the isomotive DEP force, all cells began to move at a constant speed along the centerline of the channel. At the same time, the cells also began to form small chain-like clusters, and the cell clusters continued to move while maintaining their orientation parallel to the lines of electric force. The chain-like clusters were formed by dipole–dipole interactions between neighboring cells. The velocity of single cells and cell clusters remained constant until they approached the exit of the channel where the intensity of $\nabla {E_{rms}}^{2}$ rapidly decreased.

Only the velocity of a single cell was measured when it was moving at a constant velocity. This is because the forces acting on single cells and cell clusters were different, and therefore the speed of movement was different. The cell diameter d was measured using snapshots of the movies. The representative values of the mass density of the three types of cells $\rho_{c}$ were calculated using the reported data [30,31]. The coefficient of kinetic friction between the cell and glass substrate μ was measured by sliding down cells on the tilted slide glass with a constant velocity and then noting the minimum of the tilted angle θ using μ=tanθ. The uniform movement of cells on a tilted slide glass was observed with an in-house measuring device. The slide glass was tilted slowly until the cells began to slide, and once they began to slide, the tilt angle was fixed, and movies were recorded. By analyzing movies, the constant velocity motion of the cells was confirmed and determined the μ. The values of d, $\rho_{c}$, and μ for the three types of cells used are listed in Table 1.

### 3.3. Setup and Procedure of Electrorotation

To examine the accuracy of the values of Re(β) estimated using the Creek-gap electrode device, the traditional electrorotation technique [32,33] was adopted for comparison. Electrorotation is a commonly used method to analyze the dielectric properties of cells, consisting of four identical electrodes (quadrupole electrode) that are mutually rotated by 90°. By applying AC voltages with 90° phase shifts to the quadrupole electrode, an in-plane rotating electric field is formed, and cells in the central part of the quadrupole electrode exhibit in-plane rotation. The equilibrium rotation of a single cell is established by the balance between the Stokes drag force due to fluid viscosity and the electric torque induced on the cell. The cell rotation speed $\Omega_{c}$ is related to the imaginary part of the CM factor β as follows [28]:

(14) $$Im\left(\beta \right)=\frac{2\eta }{\;\epsilon_{0}\epsilon_{f}E^{2}\;}\Omega_{c},$$

where η and E are the viscosity of the suspension medium and amplitude of the AC electric field, respectively. However, since it is very difficult to measure the frictional force against rotation on the electrode substrate surface, the rotational frictional force was included in the model as a frictional resistance with the surrounding solution.

**Figure 4:**
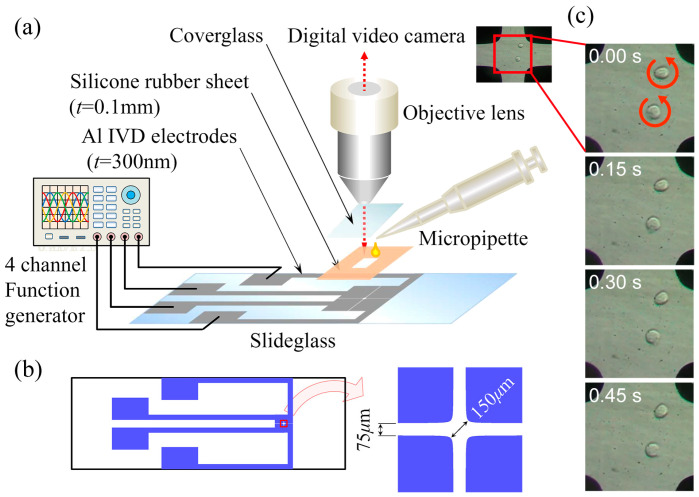
(**a**) Experimental setup of the electrorotation, consisting of a quadrupole electrode substrate, silicone rubber spacer, and cover glass. The rotating electric field was generated by a 4-channel function generator. (**b**) Electrode pattern vacuum-deposited on slide glass. The electrodes were aluminum thin films of thickness 300 nm, with the geometry of a hyperbolic function with a tip-to-tip spacing of 150 μm. (**c**) A sequence of snapshots of rotating cells taken every 0.15 s from the onset of the voltage application. Cells were rotating anticlockwise with different rotation speeds because the strength of the rotating electric field was different at different positions.

Figure 4a shows the experimental setup. The electrorotation chamber consisted of the quadrupole electrode substrate, a 15 mm square, and 0.1 mm thick silicone rubber spacer, and a cover glass on top. The silicone rubber spacer had a 2 mm square hole in the center to hold the cell suspension. The rotating electric field was generated by applying a sinusoidal voltage of 5.0 V_pp_ over the frequency range of 10–50 kHz in phase quadrature, provided by a 4-channel function generator (WF1948, NF Corporation) via four 50 Ω coaxial cables. The electrorotational behavior of the cells was monitored using an inverted microscope equipped with a digital video camera and was recorded by the camera for analysis. For the experiment, the cell suspension was pipetted into the silicone rubber enclosure, and the cover glass was gently depressed over its center to form a sealed chamber. After the cells had settled, a 5.0 V_pp_ and 20 kHz voltage was applied to the electrodes to direct cells into the central region of the quadrupole electrode by n-DEP forces repelling cells away from the electrodes. Measurements of rotation speed $\Omega_{c}$ were conducted on cells located within 50 μm of the center of the quadruple electrode to minimize the influence of the DEP force from the tips and edges of the electrode as much as possible.

The fabricated electrode substrate is shown in Figure 4b. The electrodes were made of aluminum thin films of thickness 300 nm vacuum-deposited on a slide glass using a standard photolithography method. The quadrupole electrode has the geometry of a hyperbolic function with a tip-to-tip spacing of 150 μm, which is sufficient for measurements of cells with a mean diameter of approximately 16 μm. A sequence of snapshots of rotating cells taken every 15 s from the onset of the voltage application is shown in Figure 4c. The cells were rotating anticlockwise at different speeds because the strength of the rotating electric field was different at different positions.

**Figure 5:**
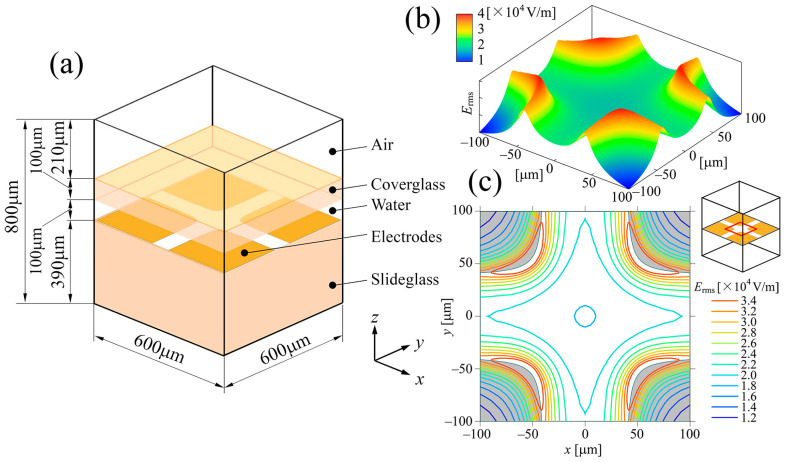
(**a**) Numerical simulation model of the 3D electric field analysis. The computational domain was a rectangular prism consisting of layers of air, cover glass, water, quadrupole electrode, and slide glass. (**b**) Distribution of $E_{rms}$ over the surface of the electrode substrate. $E_{rms}$ was obtained by time averaging the calculated electric fields E with phase angles from 0° to 90° at intervals of 5°. (**c**) Contour plots of the distribution of $E_{rms}$ over the surface of the electrode substrate. The field strength was maximal at the edge of electrode and decreased rapidly as one moved away from the edges, being minimal at the center.

Because the amplitude of the rotating electric field E in Equation (14) is difficult to measure, instead, 3D numerical prediction of the quadrupole electric field was performed to evaluate the strength of the local electric field. Figure 5a shows the numerical simulation model of the electric field analysis. The computational domain was a rectangular prism with dimensions 600 × 600 × 800 μm consisting of layers of air, cover glass, water, quadrupole electrode, and slide glass. Finer computational grids were adopted at the interfaces of the five layers and outlines of the electrodes. The governing equation for the electrostatic analysis of the electric potential ϕ was the Laplace equation:

(15) $$\frac{\partial^{2}\phi }{{\partial x}^{2}}+\frac{\partial^{2}\phi }{{\partial y}^{2}}+\frac{\partial^{2}\phi }{{\partial z}^{2}}=0,$$

which was solved using the Amaze FEM software (HiPhi v.4, Advanced Science Laboratory). An instantaneous electric potential of 5 V_pp_ across the tip-to-tip diagonal distance with phase angle 0° was assumed for boundary conditions of electrodes, namely $\phi_{0}=2.5$ V, $\phi_{0}=0$ V, $\phi_{0}=-2.5$ V, and $\phi_{0}=0$ V were imposed clockwise for four electrodes. The boundary conditions at the interfaces between air and glass and between glass and water were as follows:

(16) $$\epsilon_{a}\frac{\;\partial \phi \;}{\partial n}=\epsilon_{g}\frac{\;\partial \phi \;}{\partial n},\epsilon_{g}\frac{\;\partial \phi \;}{\partial n}=\epsilon_{w}\frac{\;\partial \phi \;}{\partial n},$$

where $\epsilon_{a}$, $\epsilon_{g}$, and $\epsilon_{w}$ are the relative electric permittivities of air, glass, and water, respectively, and ∂/∂n is the directional derivative in the direction normal to the interface. $\epsilon_{a}=1.0$, $\epsilon_{g}=6.0$, and $\epsilon_{w}=78.0$ were used for the analysis. The boundary condition over the outer surface of the computational domain was the following:

(17) $$\frac{\;\partial \phi \;}{\partial n}=0,$$

which means that the direction of the electric field was parallel to the outward normal of the outer surface of the computational domain. The number of FEM hexahedral elements used was approximately 4,200,000. Figure 5b shows the distribution of $E_{rms}$ over the surface of the electrode substrate. $E_{rms}$ was obtained by time averaging the calculated electric fields E with phase angles from 0° to 90° at intervals of 5°. The field strength was maximal at electrode edges and reduced rapidly with distance away from the edges, and it was minimal at the center of the quadrupole electrode where cells could maintain a steady rotation. Figure 5c shows contour plots of the distribution of $E_{rms}$ over the surface of the electrode substrate. The most desirable position for the accurate measurement of the cell rotation speed is the central region of the quadrupole electrode. However, placing a single cell into this restricted region was quite difficult, and dislocation of rotating cells from the central region always occurred. The discrepancy in the field strength due to the dislocation of the cell from the center was compensated for by calculating the field strength at the actual position of the rotating cell.

### 3.4. Temperature Distribution in Creek-Gap Electrode Device

In DEP-based devices, localized strong electric fields due to significant electric field line concentration at the edges of electrodes are generated even when the applied voltage is relatively low. Such a localized strong electric field leads to the generation of Joule heat [34,35,36,37] and dielectric loss heat [38], resulting in a temperature rise in devices. Temperature rises in DEP devices may affect cell viability. The Joule heat $Q_{J}$ and dielectric loss heat $Q_{d}$ are defined as follows:

(18) $$Q_{J}=\sigma_{f}{E_{rms}}^{2},Q_{d}={{\epsilon_{0}\;\epsilon }_{f}}^{\prime \prime }\omega \;{E_{rms}}^{2},$$

respectively, where ${\epsilon_{f}}^{\prime \prime }$ is the imaginary part of the complex relative permittivity of the suspension medium. To evaluate the temperature rise and distribution in the voltage-loaded Creek-gap electrode device, micro LIF thermometry using a Rhodamine B fluorescent dye was adopted.

**Figure 6:**
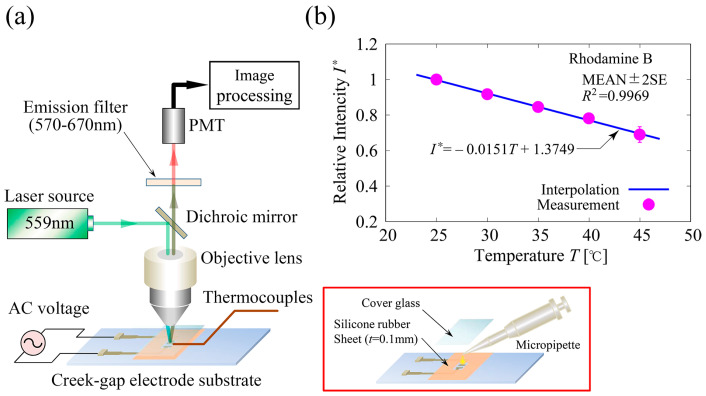
(**a**) Experimental setup of the temperature measurement system using micro LIF thermometry. A Rhodamine B solution of 20 μM was used with electric conductivity $\sigma_{f}=40$ mS/m. Fluorescence images were acquired using a confocal laser-scanning microscope. The test section filled with the Rhodamine B solution using a micropipette in the silicone rubber enclosure with a cover glass on the top was placed on the stage of the microscope. A sinusoidal AC voltage was applied with V=20.0 V_pp_ and f=40 kHz. The initial temperature of the solution was measured using T-type thermocouples. (**b**) Calibration curve of the translation of fluorescence intensity into temperature. Data for calibration were obtained using a fluorescence microplate reader. Fluorescence intensities were measured by changing the temperature in the compartment of the reader from 25 to 45°. The solid line is linear best fit of the measured data with determination coefficient $R^{2}=0.9969$ used for the translation.

Figure 6a shows the experimental setup of the temperature measurement system. The Rhodamine B (Sigma 83689) had a peak excitation wavelength of 553 nm and a peak emission wavelength of 627 nm and was dissolved in a mannitol solution (300 mM, $\sigma_{f}=40$ mS/m) to a final concentration of 20 μM. For the preparation of the sample solution, a small amount of high concentration Rhodamine B solution was added to the mannitol solution to achieve a concentration of 20 μM, and finally the electric conductivity of the solution was prepared to $\sigma_{f}=40$ mS/m using the culture media. Acquisition of 2D images of Rhodamine B fluorescence was conducted using a confocal laser-scanning microscope (Olympus, FV1000). The test section was filled with the Rhodamine B solution in the silicone rubber enclosure with a cover glass on the top and was placed on the stage of the microscope. A sinusoidal AC voltage of V=20.0 V_pp_ was applied at a fixed frequency of f=40 kHz. The initial temperature of the solution was measured using T-type thermocouples. Excitation light with a wavelength of 559 nm was supplied by a light-emitting diode. The laser light sheet was focused on the surface of the electrode substrate, and then the 2D fluorescence image was detected with PMT through the dichroic mirror and band-pass filter (570–670 nm; peak approximately 620 nm). The calibration curve of the translation of the fluorescence intensity to temperature is shown in Figure 6b. Calibration data were obtained using a fluorescence microplate reader (Beckman Coulter, DTX–880) with 535/625 nm ex/em filter settings. The fluorescence intensities of the Rhodamine B solution in a 96-well microtiter plate were measured by changing the temperature in the compartment of the microplate reader from 25 to 45°. Twenty-four measurements were performed for data analysis. Linear regression analysis was performed on the measured data, and the resulting linear best fit of the measured data with the determination coefficient $R^{2}=0.9969$ was used to obtain the temperature from 2D fluorescence images.

### 3.5. Statistical Analysis

Results were statistically analyzed by one-way analysis of variance (ANOVA) followed by the Tukey–Kramer post-hoc test to determine differences between the groups. Values are expressed as MEAN±2SE. The criterion for statistical significance was set at p ≤ 0.05.

## 4. Results and Discussion

### 4.1. DEP Force Measurement

**Figure 7:**
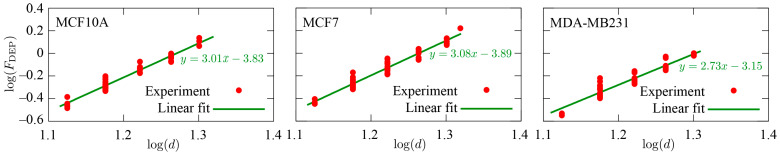
Relationship between the cell diameter d and strength of the DEP force, $F_{DEP}$, for three types of cells. Cell diameter was measured, and $F_{DEP}$ was calculated using Equation (4). These results demonstrate that the $F_{DEP}$ is proportional to the cubic of cell diameter d, i.e., ($F_{DEP}{\sim d}^{3}$), and are also experimental verification of Equation (1) proved by the use of the Creek-gap electrode device.

The measured cell velocities v, diameters d, and frictional coefficients μ were used to evaluate the magnitude of the DEP force, $F_{DEP}$ ($=F_{s}+F_{f}$), in Equation (5). The calculated values of $F_{DEP}$ were found to fall within the range of 0.5–2.0 pN, and this range of the order of $F_{DEP}$ was consistent with that necessary to effectively manipulate mammalian cells [39,40,41]. The dependence of the $F_{DEP}$ on the cell diameter was further examined to validate the correctness of the device operation and measurement accuracy. Figure 7 shows that the relationship between d and $F_{DEP}$ is correlated as follows:

(19) $$F_{DEP}\sim d^{3}$$

regardless of the cell type. The results demonstrate that the $F_{DEP}$ is proportional to the cubic of cell diameter $d^{3}$, which experimentally verifies Equation (1).

### 4.2. Re(β) Variation with Frequency

**Figure 8:**
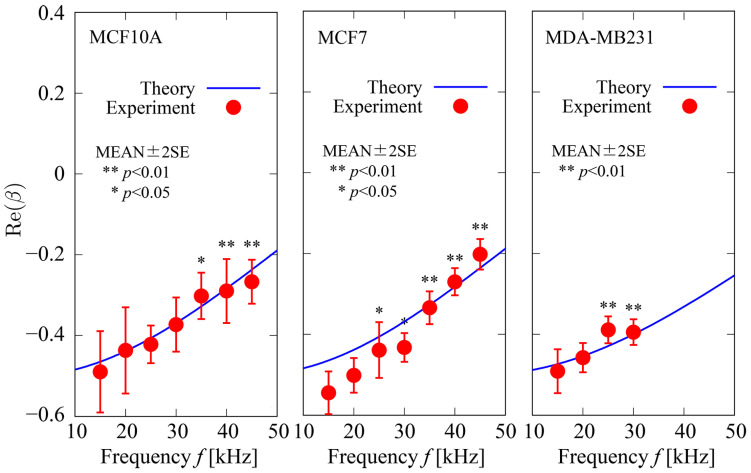
Dependence of Re(β) on field frequency f for three types of cells. Circles with bars represent MEAN±2SE of Re(β) obtained using Equations (5)–(7). The number of cells used for analysis was n=10 for each data point. Velocity measurement was performed with the frequency range of f≈ 15–45 kHz; however, cell velocities could not be measured for MDA−MB231 for frequencies f⪆35 kHz because the magnitude of DEP forces induced on cells was smaller than that of the frictional forces. Solid curves represent analytical predictions obtained using Equation (20).

The dependences of Re(β) on the field frequency f for three types of cells, MCF10A, MDA–MB231, and MCF7, are shown in Figure 8. In the figure, circles with bars represent the MEAN±2SE of Re(β) obtained using Equations (5)–(7). The number of cells used for the analysis was 10 for each data point. Velocity measurements were performed with a frequency range of f~15–45 kHz; however, cell velocities could not be measured for MDA–MB231 at frequencies f⪆35 kHz because the magnitude of the DEP forces generated on cells was smaller than that of frictional forces. Solid curves represent analytical predictions of the Re(β) correlation with f obtained from the modified form of β [28]:

(20) $$\beta =\frac{{\;\omega }^{2}\left(\tau_{f}{\tau_{c}}^{*}-\tau_{c}{\tau_{f}}^{*}\right)+j\omega \left({\tau_{f}}^{*}-\tau_{f}-{\tau_{c}}^{*}\right)-1}{{\;\omega }^{2}\left(2\tau_{f}{\tau_{c}}^{*}+\tau_{c}{\tau_{f}}^{*}\right)-j\omega \left({\tau_{f}}^{*}+2\tau_{f}+{\tau_{c}}^{*}\right)-2\;},$$

$${\tau_{c}}^{*}=\frac{\;c_{m}d\;}{\;2\sigma_{c}\;},\tau_{c}=\frac{\;\epsilon_{0}{\;\epsilon }_{c}\;}{\;\sigma_{c}\;},{\tau_{f}}^{*}=\frac{\;c_{m}d\;}{\;2\sigma_{f}\;},\tau_{f}=\frac{\;{\epsilon_{0}\;\epsilon }_{f}\;}{\;\sigma_{f}\;},$$

where $c_{m}$, $\sigma_{c}$, and $\epsilon_{c}$ are the capacitance of the cell membrane, electric conductivity, and relative electric permittivity of the model, respectively. The values of the model parameters used are listed in Table 2 [42,43,44]. The results show good agreement with the predictions.

Considering the general nature of the dielectric properties of mammalian cells, the dependence of Re(β) on f varies with the variation in the values of parameters $c_{m}$, $\sigma_{c}$, and $\epsilon_{c}$ as well as $\sigma_{f}$. In particular, at frequencies of approximately f<50 kHz, the membrane capacitance $c_{m}$ becomes dominant in determining the value of Re(β). Even for a small change in the value of $c_{m}$, the profiles of f–Re(β) curves in the range of low frequencies (approximately <100 kHz) could be altered significantly. In other words, when the value of $c_{m}$ is unknown or is to be determined by some means of measurement, it can be estimated by the best fit of the theoretical f–Re(β) curves to experimental data obtained using the Creek-gap electrode. In the present study, the values of membrane capacitance $c_{m}$ for three types of cells were estimated by adopting the non-linear least square method on data obtained experimentally to determine the best fit of theoretical f–Re(β) curves to the experiment. The best fit curves were determined as follows. Because the CM factor β is also a function of $c_{m}$, Equation (20) can be expressed as follows:

(21) $$\beta =\beta \left(c_{m}\right).$$

The sum of squares of the relative residuals of Re(β) between theory and experiment, $\delta_{N}$, is defined as follows:

(22) $$\delta_{N}=\frac{\sum_{i=1}^{N}{\left[Re{\left(\beta \left(c_{m}\right)\right)}_{f_{i}}-{Re\left(\beta \right)}_{f_{i}}\right]}^{2}\;}{\sum_{i=1}^{N}{\left[{Re\left(\beta \right)}_{f_{i}}\right]}^{2}\;},$$

where $Re{\left(\beta \left(c_{m}\right)\right)}_{f_{i}}$ is the theoretical value of $Re\left(\beta \right)$ at $f=f_{i}$, calculated using Equation (20), and $Re{\left(\beta \right)}_{f_{i}}$ is the experimental value of $Re\left(\beta \right)$ obtained at $f=f_{i}$. The optimal value of $c_{m}$, which gives the minimum $\delta_{N}$, was determined by numerical simulations. For the model parameters in Equation (20), other than $c_{m}$, fixed values of $\epsilon_{c}=50.0$, $\epsilon_{f}=78.0$, and $\sigma_{f}=40$ mS/m (Table 2) were used. For the cell diameter d, the average experimental values (MEAN) for each type of cell (Table 1) were used for analysis. The resulting estimated values of $c_{m}$ for the three types of cells are listed in Table 3 with comparisons to a previous report [44]. The values of $c_{m}$ estimated in the present study were in good agreement with previously reported values. The relative errors of the estimated values to the reported values were less than 1.1%.

### 4.3. Electrorotation Experiment

**Figure 9:**
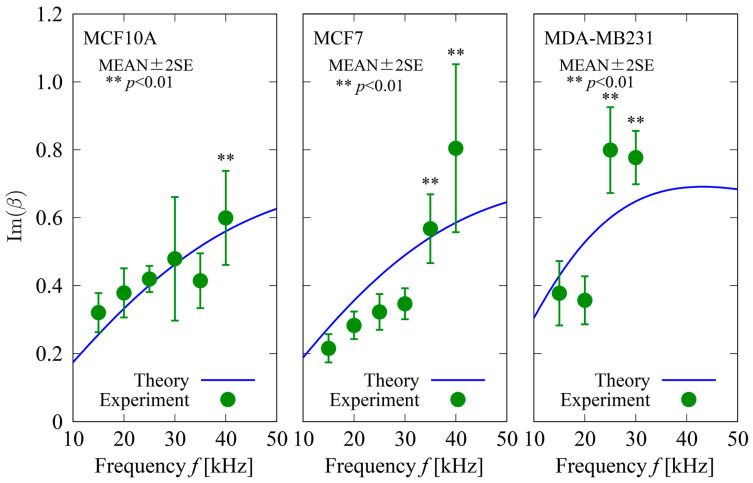
Dependence of $Im\left(\beta \right)$ on field frequency f for three types of cells. Circles with bars represent MEAN±2SE of $Im\left(\beta \right)$ obtained using Equation (14). Measured cell rotation speed $\Omega_{c}$ was used for calculations of $Im\left(\beta \right)$. The number of cells used for analysis was n=10 for each data point. Measurement of $\Omega_{c}$ was performed in the frequency range of f≅ 15–45 kHz; however, reliable data were not obtained for MDA−MB231 for frequencies f⪆35 kHz because of cell attraction and adhesion to edges of quadrupole electrodes. Solid curves represent analytical predictions obtained using Equation (20).

The dependences of $Im\left(\beta \right)$ on the field frequency f for three types of cells, MCF10A, MDA–MB231, and MCF7, are shown in Figure 9. In the figure, circles with bars represent the MEAN±2SE of $Im\left(\beta \right)$ calculated using Equation (14). The measured cell rotation speed $\Omega_{c}$ was used to calculate $Im\left(\beta \right)$. The number of cells used for the analysis was 10 for each data point. Measurement of $\Omega_{c}$ was performed in the frequency range of f~15–45 kHz; however, reliable data were not obtained for MDA–MB231 for frequencies f⪆35 kHz because of cell attraction and adhesion to the edges of the electrodes. The solid curves represent the analytical predictions obtained using Equation (20). In the calculations of the theoretical value of $Im\left(\beta \right)$, numerical predictions of the rotating electric field were used to estimate the electric field strength at locations where the cells were rotating. In a manner similar to that adopted in the previous section, the membrane capacitance of cells, $c_{m},$ can be estimated by the best fit of the theoretical $f–Im\left(\beta \right)$ curves to the experimental data. The values of $c_{m}$ for the three types of cells were estimated by adopting non-linear least square fitting on data obtained experimentally to determine the best fit of theoretical $f–Im\left(\beta \right)$ curves to the experiment. For model parameters other than $c_{m}$ appearing in Equation (20), the same values of ${\;\epsilon }_{c}$, ${\;\epsilon }_{f}$, $\sigma_{c}$, and $\sigma_{f}$ as used in the Creek-gap analysis (Table 2) were used. For the cell diameter d, average values (MEAN) for each type of cell (Table 1) were used for analysis. The resulting estimated values of $c_{m}$ for the three types of cells are listed in Table 4 with comparison to a previous report [44]. The relative errors of the estimated values to the reported values were not as small as those obtained using the Creek-gap electrode device. This is because the positions of the rotating cells in the quadrupole electrode device were not stable during the measurement. Rotating cells randomly and gradually changed their positions due to weak DEP forces induced by the nonuniform electric field generated by the electrodes. This unavoidable behavior of cells causes a variation in the rotation torque induced on cells depending on the field strength, leading to inaccurate estimation of $Im\left(\beta \right)$. To solve this problem, the introduction of a laser tweezer to fix the position of the rotating cells would be an effective choice. However, this method costs much more than using the Creep–gap electrode device. The Creek-gap electrode device does not require any means of fixing the cell position to measure $Re\left(\beta \right)$. The electrorotation method also requires a power source that can provide synchronous individual quadrupole electric potentials. The Creek-gap electrode device can accurately measure the dielectric properties of cells at a far lower cost than conventional electrorotation methods.

**Figure 10:**
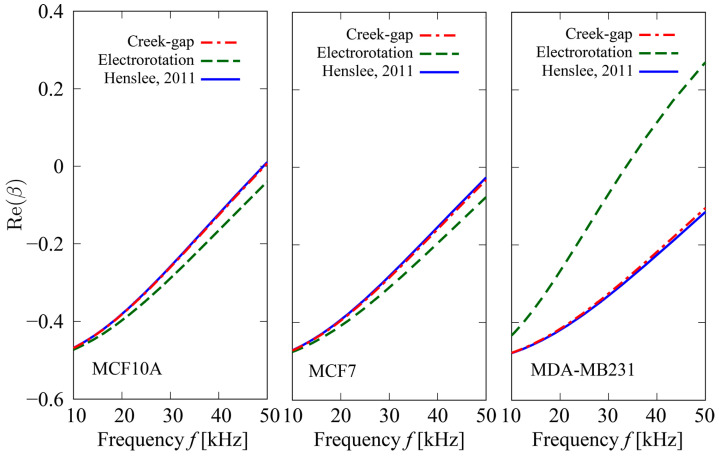
Predictions of f–Re(β) correlation for three types of cells obtained using values of cm estimated by the Creek-gap electrode and electrorotation devices, with comparison to the previous results [44]. In the calculation of the prediction curves, fixed values of model parameters were used: $\epsilon_{c}=50.0$, $\epsilon_{f}=78.0$, and $\sigma_{f}=40$ mS/m (Table 2). For the cell diameter d, mean values (Table 1) were used. Predictions obtained using the Creek-gap electrode device show excellent agreement with those of previous reports.

Figure 10 shows predictions of $f-Re\left(\beta \right)$ correlation for three types of cells obtained by using values of $c_{m}$ estimated by the Creek-gap electrode and electrorotation devices, with comparison to previous results [44]. In the calculation of the prediction curves, fixed values of model parameters were used: $\epsilon_{c}=50.0$, $\epsilon_{f}=78.0$, and $\sigma_{f}=40$ mS/m (Table 2). For the cell diameter d, mean values for each type of cell (Table 1) were used. Predictions obtained by using the Creek-gap electrode device show excellent agreement with those of previous reports.

### 4.4. Temperature Rise in the Creek-Gap Electrode Device

**Figure 11:**
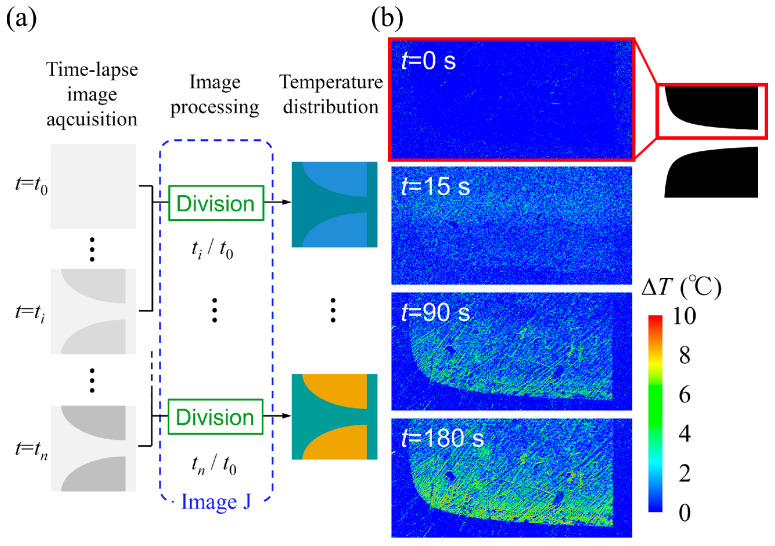
(**a**) Outline of the image-processing procedure. The captured images of Rhodamine B fluorescence were recorded as 256 level (8 bit) grayscale images having the resolution of 800×800 pixels. Sequentially captured images were processed by dividing all the captured images with the image initially captured at room temperature. Processed images were further translated into distributions of temperature rise. (**b**) Transient of the distribution of the temperature rise ∆T in the Creek-gap electrode device. Temperature over the surface of the electrode gradually rose after the onset (t=0 s) of the voltage application. However, the surface temperature did not have a uniform distribution. Regions of high temperature were seen on the edge of the electrode along the channel.

Figure 11a shows an outline of the image-processing procedure. The captured images of Rhodamine B fluorescence were recorded as 256 level (8 bit) grayscale images with a resolution of 800×800 pixels using the image-processing software of the microscope system. Sequentially captured images were processed by dividing all the captured images with the image initially captured at room temperature using the Image J software (v.1.54g, NIH). Image division was performed not merely to normalize fluorescence intensity, but also to cancel spatial irregularities in intensities of illumination and fluorescence [45]. The processed images were translated into distributions of temperature rise. Figure 11b shows the transient of the distribution of the temperature rise ΔT in the Creek-gap electrode device. The temperature over the surface of the electrodes gradually increased after the onset of the voltage application. However, the surface temperature did not exhibit a uniform distribution. Regions of high temperature were observed at the edges of the electrodes along the channel. Because the rate of heat generation is defined by Equation (18), a greater increase in temperature was observed along the outline of the electrode, where the strongest electric field appeared. The temperature rise ΔT was approximately several degrees Celsius at time t=180 s over the surfaces of the electrodes, while the temperature in the channel barely changed. However, the temperature rise of the solution would be most pronounced slightly above the channel due to the presence of the electro thermal flow [46]. This implies that the heat generation in the Creek-gap electrode device caused by the electric current in the suspension medium barely affects the viability of cells moving along the centerline of the channel. However, when the room temperature is 30 degrees Celsius or higher, the cells in the channel could be damaged by heat generation.

## 5. Concluding Remarks

The establishment of fast and accurate methods of estimating the real number part of the CM factor Re(β) of biological cells is crucial for the development and progress of high-throughput dielectrophoretic cell separation technologies. In the present study, a Creek-gap electrode device was developed to realize fast and accurate estimation of Re(β). As a notable feature, the Creek-gap electrode device could provide isomotive motion to cells along its channel in which velocities of moving cells are measured. The magnitude of the dielectrophoretic force induced on a single cell was estimated by measuring the cell velocity. Values of Re(β) were able to be estimated using the calculated DEP forces and the value of the gradient of the square of the electric field $\nabla {E_{rms}}^{2}$ obtained by numerical simulation. Estimated values of Re(β) were validated by comparison with those obtained by traditional electrorotation experiments and with those of previous studies. The results demonstrate that Re(β) correlations with the field frequency f were in good agreement with those of previous studies. Moreover, the temperature rise in the device, which could negatively affect the physiological functions of cells, was also measured using the micro LIF method and was found to be approximately several degrees Celsius at most. This temperature rise range may have a minor effect on cell function. The temperature measurement performed in this study was only a rough estimate of the order of temperature rise in the device due to Joule heating in order to consider the degree of adverse effect on cells, and we believe that the exact measurement of the temperature rise and the transient variation in temperature distribution are future issues. Thus, the Creek-gap electrode device could be a powerful tool for the fast and accurate estimation of Re(β). However, the limitation of the use of the Creek-gap electrode device is that Re(β) can only be estimated within the frequency range in which cells exhibit n–DEP characteristics. It is also difficult to estimate Re(β) at frequencies near the crossover point, where Re(β) is zero. To overcome this limitation, developing an inverted-type (up-side down) Creek-gap electrode device would be an effective solution.

## Figures and Tables

**Table 1:** Diameter d, mass density $\rho_{c}$, and coefficient of kinetic friction μ of three types of cells. The values of d and μ are measured values. The values of $\rho_{c}$ were calculated using reported data [30,31].

	d (MEAN±2SE) ^a^	$\rho_{c}$	μ (MEAN±2SE) ^b^
MCF10A	15.8 ± 0.52 µm	1.18 g/cm^3^	0.12 ± 0.01
MCF7	16.8 ± 0.58 µm	1.08 g/cm^3^	0.32 ± 0.01
MDA-MB231	16.5 ± 0.48 µm	1.12 g/cm^3^	0.26 ± 0.01

^a^ determined by measurement of 50 cells for each type of cell. ^b^ determined by measurement of 10 cells for each type of cell.

**Table 2:** Relative permittivity of cell cytosol $\epsilon_{c}$, relative permittivity of suspension medium $\epsilon_{f}$, conductivity of cell cytosol $\sigma_{c}$, conductivity of suspension medium $\sigma_{f}$, and membrane capacitance $c_{m}$ of three types of cells used for analysis.

	MCF10A	MCF7	MDA−MB231
$\epsilon_{c}$ [43]	−	50	−
$\epsilon_{f}$	−	78.0	−
$\sigma_{c}$ (mS/m) [43]	1000	750	480
$\sigma_{f}$ (mS/m)	−	40	−
$c_{m}$ (×10^−2^ F/m^2^) [44]	1.94	1.86	1.63

**Table 3:** Values of membrane capacitance cm for three types of cells, estimated by the best fit of experimental data obtained using the Creek-gap electrode device, data from Ref. [44], and relative errors of the estimated values to the reference values.

	$c_{m}$$\;(\times 10^{-2}$ F/m^2^)		Relative Error (%)
	Present	Ref. [44]	
MCF10A	1.93	1.94	0.52
MCF7	1.84	1.86	1.08
MDA−MB231	1.66	1.63	1.84

**Table 4:** Values of membrane capacitance $c_{m}$ for three types of cells, estimated by the best fit of data of electrorotation experiment, data from Ref. [44], and relative errors of the estimated values to the reference values.

	$c_{m}$ ($\times 10^{-2}$ F/m^2^)		Relative Error (%)
	Present	Ref. [44]	
MCF10A	1.79	1.94	7.7
MCF7	1.71	1.86	8.1
MDA−MB231	2.95	1.63	81.0

## Data Availability

Data are contained within the article.
